# Epidemiological Characteristics and Environmental Risk Factors of Severe Fever with Thrombocytopenia Syndrome in Hubei Province, China, from 2011 to 2016

**DOI:** 10.3389/fmicb.2017.00387

**Published:** 2017-03-08

**Authors:** Tang Wang, Xin-Lou Li, Man Liu, Xiao-Jia Song, Hao Zhang, Yu-Bin Wang, Bao-Pin Tian, Xue-Sen Xing, Shi-Yue Li

**Affiliations:** ^1^School of Health Sciences, Wuhan UniversityWuhan, China; ^2^Center for Disease Control and Prevention of Aerospace SystemBeijing, China; ^3^Hubei Provincial Center for Disease Control and PreventionWuhan, China; ^4^Shiyan Center for Disease Control and PreventionShiyan, China; ^5^Yichang Center for Disease Control and PreventionYichang, China

**Keywords:** epidemiological, dynamic, human infection, severe fever with thrombocytopenia syndrome, transmission

## Abstract

Severe fever with thrombocytopenia syndrome (SFTS) is a tick-borne viral disease affecting hundreds of people in China each year. To better understand the epidemiological characteristics and environmental risk factors associated with the incidence of SFTS in Hubei Province, China, we conducted a retrospective epidemiological study and risk assessment of SFTS from 2011 to 2016. Although, the incidence and epidemic areas of SFTS are increasing, the fatality rate has decreased. Elderly farmers are the population most commonly infected with SFTS virus between May and July in the northeast Hubei Province, which seems to be consistent with local agricultural activities and the seasonal abundance of ticks. Spatial scanning showed that regions bordering with Xinyang City, Henan Province accounted for most of the SFTS cases in Hubei Province, and there was a significant association of SFTS incidence with temporal changes in the climate within these clusters. Multivariate modeling analysis identified density of cattle, rain-fed cropland, built-up land, temperature, and relative humidity as independent risk factors for the distribution of SFTS. Future epidemiological and serological studies are warranted to elucidate the dynamics and immunity patterns of local SFTS disease and to optimize interventions.

## Introduction

Severe fever with thrombocytopenia syndrome (SFTS) is an emerging infectious disease that was first reported in China in 2009 (Yu et al., 2011). The causative virus (SFTSV) was isolated from an infected human (Yu et al., 2011), and since then, confirmed SFTSV infected patients has been detected in 19 provinces of mainland China (Liu et al., 2015) as well as in other countries such as Japan, South Korea, USA, and the United Arab Emirates (McMullan et al., 2012; Kim et al., 2013; Takahashi et al., 2014). Most cases were reported in China, with a high fatality rate of 12–30% (Yu et al., 2011).

In China, the most highly affected region is in central China, where over one-third of the total cases have been reported. Hubei Province is located in central China, and was ranked third in terms of the total number of SFTS cases in the country (Liu et al., 2015), and most patients were originated from Dabieshan mountain and the surrounding foothills. In fact, as early as 2005, a cluster infection of seven patients with clinical manifestation of fever and thrombocytopenia was first reported in Hubei Province, one of whom died. However, owing to the lack of laboratory evidence, such patients were reported as having “probable human granulocytic anaplasmosis.” However, in 2010, a novel virus, bunyavirus named HB29, was discovered and successfully isolated from acute phase serum of one such patient in Hubei Province (Yu et al., 2011).

Previous studies have described the epidemiological features of human infection with SFTSV in the city of Xinyang, in Henan Province (Huang et al., 2014; Liu et al., 2014), Zhejiang Province (Zhang et al., 2014), and in Shandong Province (Du et al., 2014), and the environmental factors (Du et al., 2014; Liu et al., 2014, 2015) that are associated with the transmission and dynamics of the SFTS. Although, the SFTSV was identified in Hubei Province for the first time, the epidemiological characteristics and environmental risk factors of SFTS in Hubei Province remain unclear. Here, we conducted a systematically study to understand the epidemiological characteristics of cases of SFTS, to explore spatial, temporal, climate, and ecological risk factors for human SFTS cases, and to identify the environmental risk factors that may be associated with human infections, from 2011 to 2016 in Hubei Province.

## Materials and Methods

### Study Area

**FIGURE 1:**
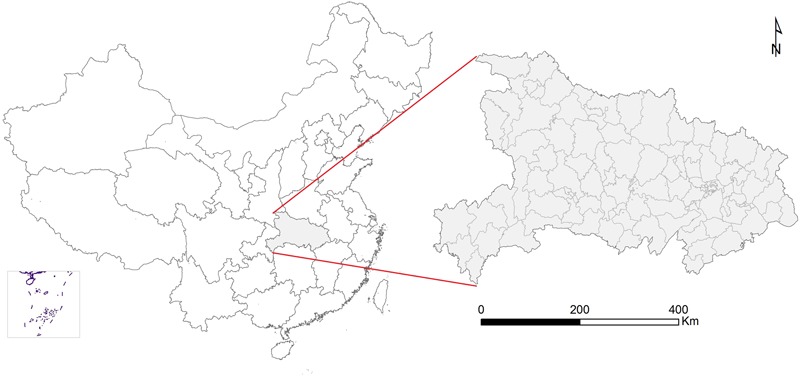
**Spatial distribution of Hubei Province and its location in China**.

Hubei Province is located in central China between 107°56’-116°58’E and 27°58’-34°03’N, and includes 1424 administrative townships of 102 prefectures (**Figure 1**), with a total population of 57,000,000, and a total area of 186,000km^2^. The region is characterized by its distinct natural landscapes, with the northern region mainly comprising plains and the southern region stretching across the Dabieshan mountain range.

### Case Definitions

According to the national guidelines for SFTS diagnosis (The Ministry of Health of People’s Republic of China, 2010), a probable SFTS case is defined as a patient with acute fever, thrombocytopenia and/or leucopenia, and a confirmed case is defined as a probable case with laboratory evidence meeting one or more of the following criteria: (1) a positive SFTSV culture, (2) a positive result for SFTSV RNA by molecular detection, and (3) seroconversion or >4-fold increase in specific antibody to SFTSV between acute and convalescent serum samples. All laboratory assays were conducted in Hubei Provincial Center for Disease Control and Prevention.

### Data Collection and Management

Data on probable and confirmed SFTS cases from January 1, 2011 to December 31, 2016 were extracted and collected from the China Information System for Diseases Control and Prevention (CISDCP) from the Centre for Disease Control and Prevention of Hubei Province (Hubei CDC). Demographic information, including age, sex, occupation, onset date of symptoms, and residential township, were collected.

A digital township-level map of Hubei Province was obtained from the Data Sharing Infrastructure of Earth System Science^1^. Data regarding the agro-ecological, environmental, and meteorological variables, including population density, land cover, elevation, and monthly temperature, relative humidity, and precipitation, were also collected to identify the potential landscape elements contributing to the dynamic presence of SFTS. The demographics and density of the human population at the township-level were obtained from the National Bureau of Statistics of China. Land cover was extracted from “GlobCover 2009 land cover map^2^”. Percentage coverage of irrigated cropland, rain-fed cropland, forests, shrubs, grasslands, built-up land, and water bodies were extracted and summarized at the township level in ArcGIS 9.3 software (ESRI Inc., Redlands, CA, USA)^3^. Elevation data were extracted from Global Digital Elevation Data Products^4^. Meteorological data of monthly temperature, relative humidity, and precipitation were collected from the Chinese Academy of Meteorological Sciences^5^.

Each confirmed case was geo-referenced and linked to a township-level map of Hubei Province according to their residence at the time of symptom onset using geographic information system (GIS) technologies. To characterize the epidemiological features of SFTS cases in Hubei during 2011 to 2016, the demographic, temporal, and spatial distribution was assessed. Epidemic curve was created by plotting the monthly number of confirmed cases according to their symptom onset. The total number of cumulative confirmed SFTS cases for each sex and the annual incidence over different age groups were calculated and plotted. The spatial distributions of confirmed SFTS cases for each year were created by mapping the cumulative confirmed SFTS cases to explore the spatial dynamics of occurrence of SFTS.

### Statistical Analysis

Panel Poisson regression was performed at the township-level, with a temporal resolution of 1 year to explore the association between the dynamics of SFTS, and environmental and meteorological factors. Potential environmental factors, such as percentage coverage of forest, shrub land, cropland, and grassland for each township, were included as co-variables in the analysis. Meteorological factors that were included in the analysis included temperature, relative humidity, and precipitation for the 4 months of the year when SFTS rates are at their highest. The incidence rate ratio (IRR) in response to the change of a variable by a given amount (10% for the percentage coverage of forest, shrub land, cropland and grassland, and 100 mm for precipitation), was used to determine the impact of each variable on disease incidence. The 95% confidence interval (CI) and corresponding *p*-values were estimated after correcting for overdispersion. The possible interactions between each covariate were also included in the analysis. Similarly, continuous variables were also presented as categorical results in order to allow inspection of the data, and determine if assumptions regarding continuous variables were justified. If significant, non-linear associations between presence of SFTS infections and the variables were found, they were incorporated into a polynomial regression analysis. Models were also optimized by comparing the 2-log likelihood, and goodness of fit with continuous, or categorical variables, respectively, which had a non-liner association with the presence of SFTS cases when they were either added or removed from the models. However, no better models were found when categorical variables were included.

### Ethics Statement

The data of SFTS patients were extracted and collected from the China Information System for Diseases Control and Prevention (CISDCP) which was authorized and managed by the National Health and Family Planning Commission. Informed consent was obtained from all individual participants included in the study.

## Results

**FIGURE 2:**
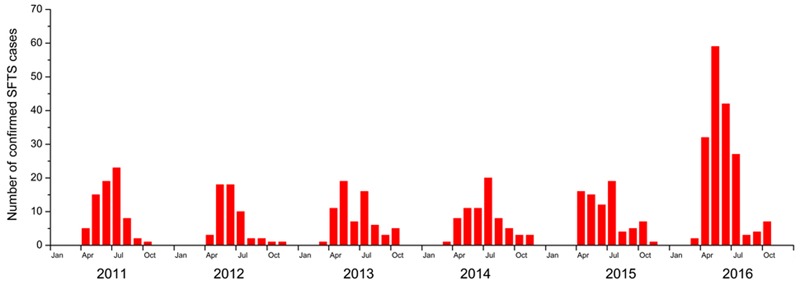
**Number of severe fever with thrombocytopenia syndrome cases in Hubei Province per month, from 2011 to 2016**.

A total of 521 laboratory-confirmed SFTS cases were reported during the study period, 44 (8.45%) of which were fatal. Of the 521 confirmed SFTS cases, 278 (53.4%) were female and 437 (83.9%) were farmers. The mean age was 59.3 years, ranging from 23 to 87 years. Of the 44 fatal SFTS cases, 16 (36.4%) were female, with an average age of 63.5 years, ranging from 42 to 86 years. The confirmed number of SFTS cases per year from 2011 to 2015 was 73, 55, 68, 70, and 79, respectively, while the 176 confirmed cases reported in 2016 equals to more than a two-fold increase compared to the previous years. From 2011 to 2016, the confirmed cases showed an annual upward trend, with the number of cases increasing in April each year, and peaking between May and July (**Figure 2**). However, no confirmed cases were reported after November. The SFTS epidemic curve therefore revealed a seasonal pattern, with 83.7% cases occurring between May and July (**Figure 2** and **Table 1**).

**Table 1:** Summary of epidemiological characteristics of severe fever with thrombocytopenia syndrome in Hubei Province, from 2011 to 2016.

Characteristics	Total cases (*n* = 521)	Deaths (*n* = 44)
Female, No. (%)	278 (53.4%)	16 (36.4%)
Age, mean ± SD	59.3 ± 11.3	63.5 ± 11.1
Farmers (%)	437 (83.9%)	41 (93.2%)
Temporal distribution		
2011	73	13
2012	55	11
2013	68	8
2014	70	9
2015	79	2
2016	176	1
Epidemic peak, No. (%)	May to July, 436 (83.7%)	May to July, 31 (70.5%)
Spatial distribution		
Affected townships, No. (%)	164 (11.5%)	33 (2.3%)

**FIGURE 3:**
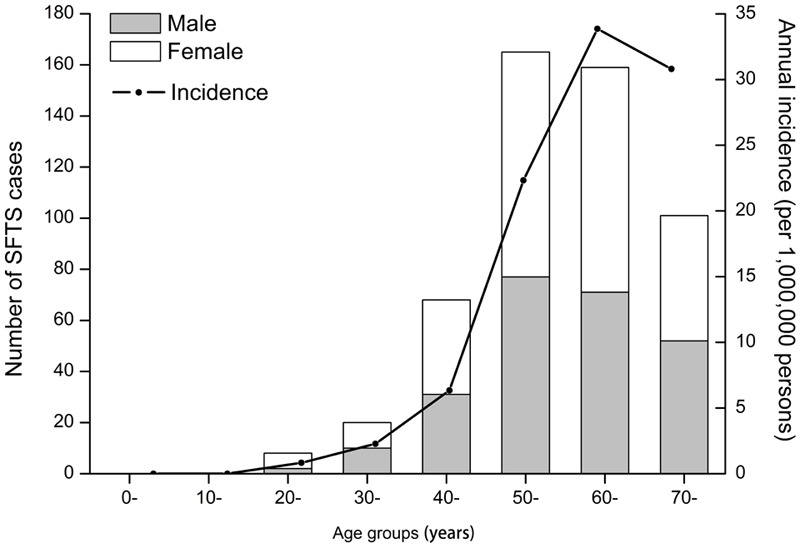
**Age and sex distribution of severe fever with thrombocytopenia syndrome cases in Hubei Province, from 2011 to 2016.** The black bar represents the number of male cases and the white bar represents the number of female cases over different age groups. The line represents the annual incidence over different age groups.

**FIGURE 4:**
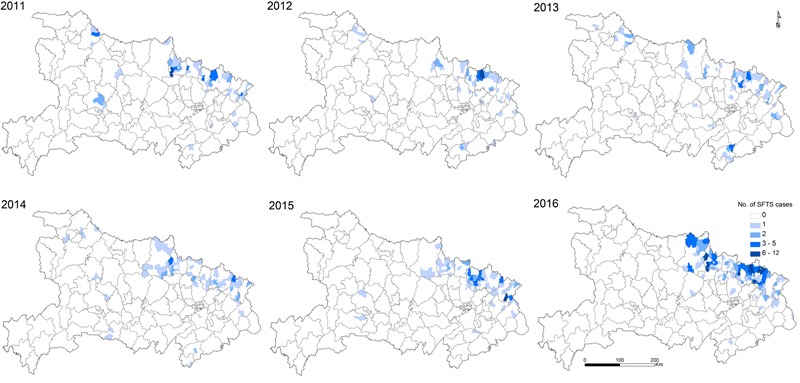
**Spatial distribution of severe fever with thrombocytopenia syndrome cases with an overlapping the map of different types of land cover in Hubei Province, from 2011 to 2016**.

**FIGURE 5:**
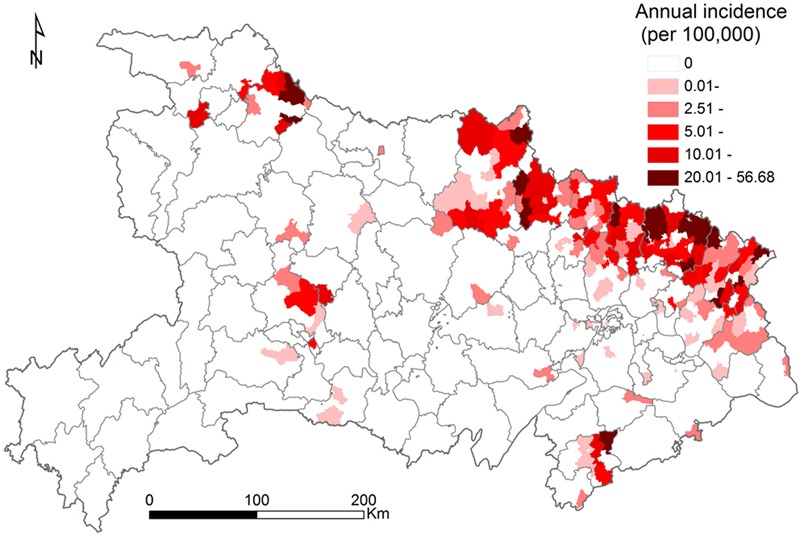
**Annual incidence of severe fever with thrombocytopenia syndrome at the township level in Hubei Province, from 2011 to 2016**.

The confirmed SFTS cases were distributed across 164 townships in 15 cities, covering 11.5% of the total area of Hubei Province. The annual incidence over different age groups demonstrated that the incidence increased with increasing age (χ^2^ test, *p* < 0.001) with people over 60 years having the highest incidence in our study (**Figure 3**). As shown in **Figure 4**, the distributions of confirmed SFTS cases were sporadic in 44 townships in 2011, and in 40, 49, 57, and 54 townships in 2012, 2013, 2014, and 2015, respectively. However, confirmed SFTS cases were more clustered in the northeast Hubei Province in 2016, i.e., in the cities of Suizhou, Xiaogan, and Huanggang border Xinyang City of Henan Province. The annual incidence varied tremendously at the township-level, ranging from 0 to 56.68 per 100,000 people, with an average of 0.97 per 100,000 people (**Figure 5**). Of 1424 townships in Hubei Province, 164 townships reported confirmed cases. Of these 164 townships with confirmed cases, 50 had an annual SFTS incidence of more than 10.00 per 100,000 people (**Figure 5**).

Panel Poisson regression analysis showed that the presences of SFTS was significantly associated with the density of both cattle and human population; the percentage coverage of irrigated croplands, rain-fed cropland, and built-up land; and temperature, relative humidity, and precipitation by univariate analysis (**Table 2** and Supplementary Table 1). In the multivariate analysis, five variables including density of cattle, percentage coverage of rain-fed cropland, percentage coverage of built-up land, temperature, and relative humidity were found to be independent risk factors for the presences of SFTS in Hubei Province at the township-level. The adjusted IRRs for density of cattle, percentage coverage of rain-fed cropland, percentage coverage of built-up land, temperature, and relative humidity were 2.03, 0.71, 0.59, 0.63, and 1.72, respectively.

**Table 2:** Summary of landscape elements contributing to the spatial dynamic of severe fever with thrombocytopenia syndrome in Hubei Province, from 2011 to 2016.

Variables (Unit)	Univariate analysis		Multivariate analysis	
	Crude IRR (95% CI)	*P-*value	Adjusted IRR (95% CI)	*P-*value
Density of cattle (continuous, 100 heads per km^2^)	5.63 (3.06–10.38)	<0.001	2.03 (1.38–3.00)	<0.001
Density of goat (continuous, 100 heads per km^2^)	1.57 (0.63–3.93)	0.338		
Density of human population (continuous, 100 persons per km^2^)	0.98 (0.98–0.99)	<0.001		
Percentage coverage of forest (continuous, 10%)	1.10 (0.94–1.28)	0.226		
Percentage coverage of irrigated cropland (continuous, 10%)	1.30 (1.23–1.38)	<0.001		
Percentage coverage of rainfed cropland (continuous, 10%)	0.70 (0.65–0.75)	<0.001	0.71 (0.66–0.76)	<0.001
Percentage coverage of grassland (continuous, 10%)	1.02 (0.83–1.27)	0.82		
Quadratic percentage coverage of grassland (continuous)	0.99 (0.96–1.02)	0.433	
Percentage coverage of built-up land (continuous, 10%)	0.62 (0.52–0.74)	<0.001	0.59 (0.50–0.69)	<0.001
Quadratic coverage of built-up land (continuous)	0.96 (0.95–0.98)	<0.001		
Temperature (continuous, 2°C)	0.78 (0.66–0.92)	0.003	0.83 (0.71–0.97)	0.022
Relative humidity (continuous, 10%)	1.89 (1.29–1.77)	0.001	1.72 (1.18–2.50)	0.005
Precipitation (continuous, 100 mm)	1.14 (1.05–1.23)	0.001		
Quadratic precipitation (continuous)	1.04 (1.01–1.07)	0.001		
Elevation (continuous, 100 m)	0.95 (0.86–1.06)	0.347		

## Discussion

Our study systematically analyzed the epidemiologic features of SFTS in Hubei Province from 2011 to 2016, which is the third-most SFTS-affected region in China. The average annual incidence of SFTS is 0.94 per 100,000 people, with an average fatality of 8.4% cases. More than 80% SFTS cases were observed in farmers, and people who were older than 60 years had the high incidence of the disease. We identified a single peak pattern of SFTS cases from May to July in Hubei Province each year. The density of cattle, rain-fed cropland, built-up land, temperature, and relative humidity were significantly associated with SFTSV infection.

Between 2011 and 2016, a total of 521 laboratory-confirmed and 1378 probable cases have been reported. Although the number of SFTS cases increased annually, only 27.4% of cases were laboratory-confirmed, which is significantly lower than the average of 51.4% of laboratory-confirmed cases reported in China. This discrepancy may due to the limited capacity of laboratory detection, especially in Hubei Province. However, the number of laboratory-confirmed SFTS cases were significantly increased in 2016 under the strengthen of laboratory detection and reporting in Hubei Province. Therefore, techniques for laboratory detection should be enhanced in future. Similar to previous studies (Ding et al., 2014; Liu et al., 2015; Sun et al., 2016), there was a high incidence of SFTS among people over the age of 60 years, with more female patients compared with male patients, which may correlate with their patterns of exposure owing to their work activities that include working in fields, or with cattle. The epidemic of SFTS showed a single peak between May and July, which is consistent with the distribution across the country (Liu et al., 2015), but a slight difference was observed in Zhejiang Province where peak was identified between May and August (Sun et al., 2014), and correlated with the activity of *Haemaphysalis longicornis*, which is highly active in this region over this time period (Tan et al., 2013).

Since the identification of SFTS cases in Hubei Province, epidemic areas of SFTS have expanded to include 15 cities between 2011 and 2016, one possible explanation is the intensive training of medical staff for public health initiatives, disease monitoring, and improved detection capacity of the pathogen. Our geographic analysis showed that the area with highest incidence of SFTS is mainly across the northeast Dabieshan mountain, which had highly disseminated cases. Among the cities located in Dabieshan Mountain, Huanggang, Suizhou, and Xiaogan had the largest number of cases, of which Suizhou City had the highest incidence, followed by Huanggang City. It is notable that these regions border Xinyang City of Henan Province, which is the most severely affected region where approximately 98.78% of the total cases in Henan Province were reported.

Ticks have been recognized as the vectors involved in SFTSV transmission to humans. People who live in mountainous or hilly rural areas were suggested to be a high-risk population (Li, 2011; Yu et al., 2011; Liu et al., 2015). It has been demonstrated that *H. longicornis* is the main host and vector for SFTSV transmission (Luo et al., 2015), and is the dominant species of ticks in Xinyang City. Three species ticks, namely *H. longicornis, Momisis longicornis*, and *Ixodes sinensis*, have been found in the region of Huanggang, and Suizhou Cities, where *H. longicornis* is also the dominant species (Fang et al., 2015), suggesting a possible epidemiological correlation between the high incidence in Cities located in Dabieshan Mountain, and the high incidence of SFTS in Xinyang City of Henan Province. Previous studies have also showed that the SFTSV infections were highly correlated with the tick density curve, and the peak of tick density occurred earlier than the peak of human infection cases (Liu et al., 2012; Du et al., 2014). The growth and reproductive cycles of ticks is closely related to environmental factors, such as climate and landform (Brownstein et al., 2003); therefore, we analyzed environmental factors that may be associated with SFTSV infection, and found that the density of cattle, percentage coverage of rain-fed cropland, percentage coverage of built-up land, temperature, and relative humidity were associated with the presence of SFTS infection in Hubei at the township level. These findings may help improve the understanding the spatial dynamics of SFTSV across a large geographic region. An association between the spatial distribution of SFTSV infection, and the density of cattle was observed. Previous study have showed that the density of goats were associated with the dynamics of SFTS (Liu et al., 2012), but no significant association was identified in present study, which could be partially explained by the fact that cattle are more commonly raised for agriculture activities compared to goats. The percentage coverage of rain-fed cropland was significantly associated with the occurrence of SFTS in Hubei Province (**Table 2**); however, this differed from previous findings in Xinyang (Liu et al., 2012), which might reflect that rain-fed cropland is mostly distributed in plains rather than in mountainous regions. Percentage coverage of built-up land is greater in urban areas, which also demonstrates that SFTSV is mainly distributed in non-urban areas. Temperature and relative humidity may also play important role in the dynamics of SFTS infection in Hubei because they have an effect on the life cycle of ticks, considered the main vectors of SFTSV. Extremes of temperature can prohibit growth and survival of ticks, and thereby decrease the incidence of SFTS. It is likely that the geographic expansion of the tick population may contribute to the spread of SFTSV in the region. Despite these associations, future studies are required to elucidate the exact role of ticks, and possibly wild animals in the transmission of SFTSV.

There were some limitations should be addressed. First, only small portion of laboratory-confirmed cases were employed to estimate the incidence of SFTS in the present study, this may underestimate the overall incidence in Hubei Province. Second, because only laboratory-confirmed SFTS cases were included for analysis which result in subjecting to reporting bias, e.g., subclinical or mild infections with the SFTSV were not captured. Third, because there was no available data on tick distribution in Hubei Province, we could not infer the association between ticks and environmental risk factors.

## Conclusion

We characterized the epidemiologic features of SFTS cases in Hubei Province, and demonstrated that the density of cattle, percentage coverage of rain-fed cropland, percentage coverage of built-up land, temperature, and relative humidity were associated with SFTS incidence. Owing to the unavailability of any vaccines against SFTS and the fatal outcomes of the syndrome, our findings could assist in identifying high-risk areas and populations for public health officials to develop targeted surveillance, educational programs, and other interventions to reduce the disease incidence in those most at risk.

## Author Contributions

X-SX and S-YL conceived and designed the research. TW, X-LL, ML, X-JS, HZ, Y-BW, and B-PT performed the data analysis and modeling. TW and X-LL drafted the manuscript and X-SX and S-YL revised the manuscript. All authors read and approved the final manuscript.

## Conflict of Interest Statement

The authors declare that the research was conducted in the absence of any commercial or financial relationships that could be construed as a potential conflict of interest.

## Footnotes

1 http://www.geodata.cn

2 http://due.esrin.esa.int/page_globcover.php

3 http://www.esri.com/

4 http://www.gscloud.cn

5 www.cams.cma.gov.cn
