# Supplementary material for: Epidemiological Characteristics and Environmental Risk Factors of Severe Fever with Thrombocytopenia Syndrome in Hubei Province, China, from 2011 to 2016
Source: Front Microbiol. 2017 Mar 8;8:387. doi: 10.3389/fmicb.2017.00387 (PMC5340758; doi:10.3389/fmicb.2017.00387)
Supplement: Supplementary file 1 [file Table_1.doc]

**TABLE 1|**Summary of landscape elements contributing to the spatial dynamic of SFTS in Hubei Province, 2011 to 2016.

| **Variables (Unit)** | **Average yearly incidence (95%CI, per 100,000 person-years)** | **Univariate analysis** | |  | **Multivariate analysis** | |
| --- | --- | --- | --- | --- | --- | --- |
|  |  | **Crude IRR (95% CI)** | ***P* value** |  | **Adjusted IRR (95% CI)** | ***P* value** |
| Density of cattle (categorical, 100heads per km^2^) |  |  |  |  |  |  |
| < 0.1 | 0.04 (0.02-0.06) |  |  |  |  |  |
| 0.1- | 0.12 (0.08-0.17) |  |  |  |  |  |
| >0.3 | 0.31 (0.26-0.37) |  |  |  |  |  |
| Density of cattle (continuous, 100 heads per km^2^) |  | 5.63 (3.06-10.38) | < 0.001 |  | 2.03 (1.38-3.00) | < 0.001 |
| Density of goat (categorical, 100 heads per km^2^) |  |  |  |  |  |  |
| <0.01 | 0.03 (0.01-0.04) |  |  |  |  |  |
| 0.01- | 0.20 (0.15-0.25) |  |  |  |  |  |
| >0.1 | 0.29 (0.24-0.34) |  |  |  |  |  |
| Density of goat (continuous, 100 heads per km^2^) |  | 1.57 (0.63-3.93) | 0.338 |  |  |  |
| Density of human population (categorical, 100 persons per km^2^) |  |  |  |  |  |  |
| < 2 | 0.23 (0.18-0.28) |  |  |  |  |  |
| 2 - | 0.19 (0.15-0.23) |  |  |  |  |  |
| > 5 | 0.03 (0.02-0.05) |  |  |  |  |  |
| Density of human population (continuous, 100 persons per km^2^) |  | 0.98 (0.98-0.99) | < 0.001 |  |  |  |
| Percentage coverage of forest (categorical, 10%) |  |  |  |  |  |  |
| <0.04 | 0.03 (0.02-0.05) |  |  |  |  |  |
| 0.04- | 0.21 (0.17- 0.26) |  |  |  |  |  |
| >1 | 0.22 (0.18-0.28) |  |  |  |  |  |
| Percentage coverage of forest (continuous, 10%) |  | 1.10 (0.94-1.28) | 0.226 |  |  |  |
| Percentage coverage of irrigated cropland (categorical, 10%) |  |  |  |  |  |  |
| <0.01 | 0.06 (0.03-0.09) |  |  |  |  |  |
| 0.01- | 0.06 (0.03-0.09) |  |  |  |  |  |
| >2 | 0.35 (0.30-0.40) |  |  |  |  |  |
| Percentage coverage of irrigated cropland (continuous, 10%) |  | 1.30 (1.23-1.38) | < 0.001 |  |  |  |
| Percentage coverage of rainfed cropland (categorical, 10%) |  |  |  |  |  |  |
| <1.5 | 0.35 (0.29-0.41) |  |  |  |  |  |
| 1.5- | 0.11 (0.08-0.15) |  |  |  |  |  |
| >6 | 0.05(0.02-0.07) |  |  |  |  |  |
| Percentage coverage of rainfed cropland (continuous, 10%) |  | 0.70 (0.65-0.75) | < 0.001 |  | 0.71 (0.66-0.76) | < 0.001 |
| Percentage coverage of grassland (categorical, 10%) |  |  |  |  |  |  |
| <0.15 | 0.08 (0.06-0.10) |  |  |  |  |  |
| 0.15- | 0.23 (0.18-0.27) |  |  |  |  |  |
| >1 | 0.18 (0.13-0.22) |  |  |  |  |  |
| Percentage coverage of grassland (continuous, 10%) |  | 1.02 (0.83-1.27) | 0.820 |  |  |  |
| Quadratic percentage coverage of grassland (continuous) |  | 0.99 (0.96-1.02) | 0.433 |  |  |  |
| Percentage coverage of built-up land (categorical, 10%) |  |  |  |  |  |  |
| <0.03 | 0.21 (0.16-0.26) |  |  |  |  |  |
| 0.03- | 0.27 (0-.22-0.33) |  |  |  |  |  |
| >0.5 | 0.03 (0.02-0.04) |  |  |  |  |  |
| Percentage coverage of built-up land (continuous, 10%) |  | 0.62 (0.52-0.74) | < 0.001 |  | 0.59 (0.50-0.69) | < 0.001 |
| Quadratic coverage of built-up land (continuous) |  | 0.96 (0.95-0.98) | < 0.001 |  |  |  |
| Temperature (categorical, 2 ℃) |  |  |  |  |  |  |
| < 11.7 | 0.18 (0.13-0.22) |  |  |  |  |  |
| 11.7- | 0.16 (0.13-0.20) |  |  |  |  |  |
| > 12.2 | 0.15 (0.11-0.18) |  |  |  |  |  |
| Temperature (continuous, 2 ℃) |  | 0.78 (0.66-0.92) | 0.003 |  | 0.83 (0.71-0.97) | 0.022 |
| Relative humidity (categorical, 10%) |  |  |  |  |  |  |
| <7.5 | 0.17 (0.13-0.21) |  |  |  |  |  |
| 7.5- | 0.17 (0.13-0.20) |  |  |  |  |  |
| >8 | 0.09 (0.01-0.17) |  |  |  |  |  |
| Relative humidity (continuous, 10%) |  | 1.89 (1.29-1.77) | 0.001 |  | 1.72 (1.18-2.50) | 0.005 |
| Precipitation (categorical, 100mm) |  |  |  |  |  |  |
| <5.5 | 0.19 (0.15- 0.24) |  |  |  |  |  |
| 5.5- | 0.10 (0.07- 0.14) |  |  |  |  |  |
| >6.8 | 0.19 (0.15- 0.23) |  |  |  |  |  |
| Precipitation (continuous, 100mm) |  | 1.14 (1.05-1.23) | 0.001 |  |  |  |
| Quadratic precipitation (continuous) |  | 1.04 (1.01-1.07) | 0.001 |  |  |  |
| Elevation (categorical, 100m) |  |  |  |  |  |  |
| <0.4 | 0.01 (0.00- 0.01) |  |  |  |  |  |
| 0.4- | 0.24 (0.19- 0.28) |  |  |  |  |  |
| >1.8 | 0.26 (0.21- 0.32) |  |  |  |  |  |
| Elevation (continuous, 100m) |  | 0.95 (0.86-1.06) | 0.347 |  |  |  |
